# Targeted next-generation sequencing analysis in couples at increased risk for autosomal recessive disorders

**DOI:** 10.1186/s13023-018-0763-0

**Published:** 2018-01-26

**Authors:** Katalin Komlosi, Stefan Diederich, Desiree Lucia Fend-Guella, Oliver Bartsch, Jennifer Winter, Ulrich Zechner, Michael Beck, Peter Meyer, Susann Schweiger

**Affiliations:** 1 Institute of Human Genetics, University Medical Center of the Johannes Gutenberg University Mainz, Langenbeckstr. 1, 55131 Mainz, Germany; 2 Human Genetics Foundation, Sperberstr. 2, 81827 Munich, Germany; 3 Human Genetics Clinic, Department of Gynecology and Obstetrics, University Hospital, Philipps University Marburg, Baldingerstr. 1, 35043 Marburg, Germany

**Keywords:** next generation sequencing, panel diagnostics, consanguineous, carrier screening, autosomal recessive

## Abstract

**Background:**

Many of the genetic childhood disorders leading to death in the pre- or neonatal period or during early childhood follow autosomal recessive modes of inheritance and bear specific challenges for genetic counseling and prenatal diagnostics. Parents are carriers but clinically unaffected, and diseases are rare but have recurrence risks of 25% in the same family. Often, affected children (or fetuses) die before a genetic diagnosis can be established, post-mortem analysis and phenotypic descriptions are insufficient and DNA from affected fetuses or children is not available for later analysis. A genetic diagnosis showing biallelic causative mutations is, however, the requirement for targeted carrier testing in parents and prenatal and preimplantation genetic diagnosis in further pregnancies.

**Methods:**

We undertook targeted next-generation sequencing (NGS) for carrier screening of autosomal recessive lethal disorders in 8 consanguineous and 5 non-consanguineous couples with one or more affected children. We searched for heterozygous variants (non-synonymous coding or splice variants) in parents’ DNA, using a set of 430 genes known to be causative for rare autosomal recessive diseases with poor prognosis, and then filtering for variants present in genes overlapping in both partners. Putative pathogenic variants were tested for cosegregation in affected fetuses or children where material was available.

**Results:**

The diagnosis for the premature death in children was established in 5 of the 13 couples. Out of the 8 couples in which no causative diagnosis could be established 4 consented to undergo further analysis, in two of those a potentially causative variant in a novel candidate gene was identified.

**Conclusions:**

For the families in whom causative variants could be identified, these may now be used for prenatal and preimplantation genetic diagnostics. Our data show that NGS based gene panel sequencing of selected genes involved in lethal autosomal recessive disorders is an effective tool for carrier screening in parents and for the identification of recessive gene defects and offers the possibility of prenatal and preimplantation genetic diagnosis in further pregnancies in families that have experienced deaths in early childhood and /or multiple abortions.

**Electronic supplementary material:**

The online version of this article (10.1186/s13023-018-0763-0) contains supplementary material, which is available to authorized users.

## Background

Diagnosing lethal fetal disorders and rare severe childhood disorders has previously been very difficult due to the large number of potential genes, the phenotypic variability associated with many known genetic causes and the challenges of an accurate definition of prenatal and often even postnatal phenotype [1]. Recently huge advances have been made in the rapid diagnosis of newborns with congenital malformations, syndromic conditions, and inherited disorders [2, 3] using whole-exome and whole-genome sequencing in medical practice enabling precision medicine for the affected neonates and precise recurrence risks for future pregnancies. This approach, however, is still inaccessible to many families and is so far only established as an option for postnatal diagnostics.

For couples with multiple affected fetuses, most diseases result from either autosomal recessive or X-linked disorders with a 25% recurrence risk for each future pregnancy for autosomal recessive and a 50% recurrence risk for male pregnancies for maternally inherited X-linked disorders. Therefore, a genetic diagnosis showing biallelic mutations or mutations on the X-chromosome in male fetuses or children, is still the requirement for targeted carrier testing in parents, risk calculations, and prenatal and preimplantation genetic diagnosis in further pregnancies. In non-consanguineous families with only one affected child/fetus autosomal dominant disorders have to be considered in first place.

Carrier screening of healthy couples has long been restricted to a limited number of ancestry-based recessive conditions (traditional targeted and/or ancestry-based screening). Recently expanded universal carrier screening (EUCS) has been advocated, entailing a twofold expansion of long-standing (preconception) carrier screening programs: allowing the simultaneous screening of a large list of diseases (‘expanded’), and also referring to a pan-ethnic screening approach (‘universal’) [4]. However, for couples with multiple affected fetuses or lethal conditions in children most likely caused by rare or ultra-rare genetic disorders, even expanded universal carrier screening will often not reveal the underlying genetic cause, which is a prerequisite for prenatal diagnosis and informed reproductive options for the family.

Next-generation sequencing (NGS, either by targeted panels or whole exome/genome sequencing [WES/WGS]) is a powerful tool for the identification of rare gene defects including disorders with an atypical presentation of a known disease [5, 6]. However, the discrimination of harmless variants from disease causing mutations particularly if not described as disease-causing yet, is a big challenge. Identifying disease causing recessive mutations in carriers is even more difficult than identifying homozygous or compound heterozygous disease-causing mutations in affected individuals, which is also reflected in the low yield of carrier identification compared to the diagnostic yield in patients. Here we report our experiences with targeted NGS for carrier screening of autosomal recessive lethal disorders in 8 consanguineous and 5 non-consanguineous couples with one or more affected children. We searched for heterozygous variants (non-synonymous coding or splice variants) in parents’ DNAs in a set of 430 genes known to be causative for rare autosomal recessive diseases with poor prognosis, filtered for genes carrying variants in both partners, and tested for co-segregation of likely pathogenic variants in a DNA sample (where available) of affected fetuses or deceased children.

## Methods

### Patients and phenotypic characteristics

We selected 8 consanguineous and 5 non-consanguineous consecutive couples at risk for severe autosomal recessive disorders seen at the genetic counselling unit of the Institute of Human Genetics of the University Medical Center Mainz for carrier screening with targeted next-generation sequencing. The inclusion criteria included loss of at least one child before the age of 2 years due to a severe/lethal condition and/or one or more miscarriages with pathological findings in the fetus (e.g. hydrops fetalis, anencephaly, skeletal malformation, brain malformation). Of the 13 couples investigated 7 couples (Families 2, 3, 4, 5, 6, 8 and 12) had lost one child: 2 children had suspected inborn errors of metabolism, 3 children had suspected neuromuscular/neurodegenerative disorders, one child had suspected lymphohistiocytosis, and one child had a congenital malformation syndrome. Further 4 couples (Families 1, 7, 9, 11) had each lost three affected children due to severe brain malformations and epilepsy (Families 1 and 7), epileptic encephalopathy (Family 11) and multiple malformations (Family 9), respectively. Two other couples had multiple miscarriages (Families 10 and 13). In addition, five of the above mentioned couples (Families 3, 4, 6, 8, 12) had one or more miscarriages due to affected fetuses besides losing an affected child. Table 1 shows a phenotypic description of the affected children and/or fetuses.

**Table 1:** Overview of the families and variants identified by targeted next-generation sequencing analysis and exome sequencing

Family ID	Consanguinity	Phenotype in the deceased child and DD	Number/gender/age of lost child/miscarriage	Gene (OMIM)	Variant identified in the couples	Variant confirmed in deceased child	CADD/Classification of variant	Disease (OMIM) Increased risk for autosomal recessive disorder	spontaneous pregnancy with prenatal diagnosis	PGD
1	yes	intrauterine epilepsy, severe brain malformation DD: early epileptic encephalopathy	3 m + f neonatal	*CTSD* (*116840) *FTCD* (*606806)	NM_001909: c.268_269insC p.(Gln90Profs*50) mat and pat het NM_001320412: c.530G > A p.(Gly177Glu) mat and pat het	***CTSD:*** **hom**	CADD: n.a./Class 4 CADD: 25.8/Class 4	**Ceroid lipofuscinosis,** **type 10 (#610127)** **Glutamate formiminotransferase deficiency (#229100)**	two, CVS: *CTSD*: het *FTCD*: wt and het	planned
2	no	severe congenital lactic acidosis DD: pyruvate dehydrogenase deficiency	1 f neonatal	*COQ2* (*609825)	mother: NM_015697: c.1197delT p.(Asn401Ilefs*15) father: c.764C > T p.(P255L)	no	CADD: 23.7/Class 5 CADD: 29/ Class 4	**Coenzyme Q10 deficiency, primary, type 1 (#607426)**	yes, AC: *COQ2*: comp het	in progress
3	yes	SIDS, severe hypertrophic cardiomyopathy DD: FAOD, primary carnitine deficiency	1 m 5 months 1 MC	*ACADVL* (*609575)	NM_001033859: c.1274 T > C, p.(L425P) mat and pat het	***ACADVL*** **: hom**	CADD: 27.4/Class 4	**VLCAD deficiency, (#201475)**	no	planned
4	yes	hyperinflammatory syndrome with hepatosplenomegaly, suspicion of hemophagocytic lymphohistiocytosis DD: PID	1f neonatal 3 MC	*UNC13D* (*608897)	NM_199242: c.2447 + 1G, p.? donor site change) mat and pat het	no	CADD: 27.4/Class 4	**Familial hemophagocytic lymphohistiocytosis type 3 (#608898)**	no	planned
5	yes	spasticity, muscular hypotonia, epileptic encephalopathy, optic atrophy DD: Leigh syndrome, Pelizeus-Merzbacher syndrome	1 m 11 months	*BRAT1* (*614506)	NM_152743: c. 1280G > A p.(Arg427Gln) mat and pat het	no	CADD: 32/ Class 4	**Rigidity and multifocal seizure syndrome (#614498)**	prenatal diagnostics declined	planned
6	no	severe muscular hypotonia, respiratory insufficiency, seizures DD: mitochondriopathy	1 f 16 months 1 MC		none				yes: no prenatal diagnostics, healthy child	no
7	yes	severe lissencephaly type 2	1 f, 2 m neonatal	*PALLD* (*608092)	NM_001166108: Exon 1 deletion mat and pat het	***PALLD*** **: hom**	CADD: n.a./ Class 3	**No OMIM disease entry** **mouse: migration defect** [17]	no	planned
8	yes	dilated cardiomyopathy, congenital myopathy DD: mitochondriopathy	1 f 5 months 1 MC	none	none	no			yes: no prenatal diagnostics: healthy child	no
9	no	1: meningocele, hydrocephalus 2: intracerebral hemorrhage, respiratory insufficiency DD: exogenous factors	1 m neonatal 1 f neonatal 1 MC	none	none	no			no	no
10	yes	3 miscarriages with ascites, cardiomegaly, skeletal deformity, biochemical suspicion of mucolipidosis type II	4 MC (f + m)	none	none	no			no	no
11	no	early-onset severe epilepsy DD: infantile epileptic encephalopathies	1 f, 2 m 16–21 months	none	none	no			no	no
12	yes	microcephaly, IUGR, cerebellar hypoplasia, rockerbottom feet, hydronephrosis	1 f 6 months 3 MC	none	none	no			no	no
13	no	anencephaly, iu lethal DD: folate deficiency	2 MC 11th week of gestation	*APAF1* (*602233)	mother: NM_181861: c.1350C > G p.(Cys450Trp) father: c.3127C > G p.(His1043Asp)	***APAF1*** **: comp het**	CADD: 23.1/Class 3 CADD: 25.7/ Class 3	**No OMIM disease entry** **mouse model: anencephaly, neurogenesis defect** [20]	no	planned

Abbreviations: *iu* intrauterine, *f* female, *m* male, *MC* miscarriage, *het* heterozygous, *hom* homozygous, *comp het* compound heterozygous, *no:* no material available for study, *DD* differential diagnosis, *CADD score* combined annotation dependent depletion framework, *PGD* preimplantation genetic diagnosis, *PID* primary immunodeficiency, *FAOD* fatty acid oxidation defect

likely or possibly causative variants and the corresponding disease in the couples are highlighted in boldface

### Targeted NGS analysis

The selected couples were analyzed with targeted exon enrichment and NGS analysis. The MPIMG-1-Test [7], which was established at our institute in 2013, provides panel diagnostics for over 1200 genes involved in syndromic and non-syndromic forms of developmental delay and intellectual disability. 430 genes were selected from this panel for AR and XL severe childhood disorders (complete list of genes included as Additional file 1: Table S1.) as a virtual panel for evaluation of the NGS data. Although in non-consanguineous families with only one affected child/fetus autosomal dominant disorders have to be considered in first place our approach of analyzing the parents did not allow the identification of de novo dominant mutations. In those couples we aimed to exclude the rare situation of recessive diseases with a high recurrence risk. Genomic DNA samples of the unaffected parents were used to generate an Illumina Paired End pre-capture library (TruSeq Custom Enrichment Kit in accordance with the manufacturer’s protocol, Illumina, San Diego, CA, USA). Paired-end 300-bp reads were sequenced twice on an Illumina MiSeq system (Illumina, San Diego, CA, USA), 10 samples per flow cell. The selected exons were covered by an average depth of 90X, with > 95% of target bases at ≥10X.

### Data analysis

High-quality reads were aligned to the human reference genome GRCh37/hg19 by SOAP2.20. A modified version of the Medical Resequencing Analysis Pipeline (MERAP, [7]) developed at the Max Planck Institute for Molecular Genetics, Berlin, Germany, was used to check all detected variants against publicly available reference datasets as dbSNP138, the 1000 Genomes Project, the Exome Variant Server, the ExAC Browser, the OMIM catalog, the Human Gene Mutation Database (HGMD) and the ClinVar database. The following criteria were used to look for rare potentially deleterious variants: MAF < 1% in dbSNP138, ESP or ExAC Browser, or present in HGMD or ClinVar as a disease-causing variant. Only variants that were non-synonymous coding or splice-site variants including small indels and predicted to be pathogenic or to affect splicing by different prediction tools (PolyPhen, SIFT, Mutation Taster initially, later CADD framework and Human Splicing Finder) were processed further. Classification of pathogenicity was carried out according to the standards of the ACMG/AMP guidelines [8]. We searched for genes in which parents either shared the same heterozygous variant (for which the offspring could be homozygous) or had different heterozygous variants in the same gene (potentially compound heterozygous in the offspring).

### Genome-wide microarray analysis

Microarray analysis of both parents was performed in 4 non-consanguineous couples in which a heterozygous potentially deleterious variant matching the phenotype of the affected fetus/child was identified in one of the parents, and in two consanguineous couples in whom no variants were found in strong candidate genes for the phenotype. In those 6 couples genome-wide microarray analysis using the Affymetrix CytoScan® HD array (Affymetrix Inc., Santa Clara, CA, USA) comprising 2, 600, 000 probes for the detection of copy number variants (CNVs, mean distance between markers was 1,7 kb) and more than 750, 000 single nucleotide polymorphisms was performed with a resolution of 200 Kb. In addition, the presence of deletions was investigated at a higher resolution. Identified CNVs were compared with the following public databases: DECIPHER database, Online Mendelian Inheritance in Man (OMIM), UCSC, the International Standards for Cytogenomic Arrays and Ensembl database. All relevant disease-associated structural variants were functionally validated by using a quantitative real-time PCR analysis.

### Cosegregation studies

Putative deleterious heterozygous variants detected in the same gene in both partners were confirmed by Sanger sequencing and tested for cosegregation with disease in the affected offspring in those cases where DNA from the affected fetus/child was available. In one case where only a dried blood spot (filter card) was available from the deceased affected child the filter paper sample was soaked in 40 μL Tris-EDTA buffer and incubated at 37 °C overnight. Genomic DNA was isolated using the DNeasy Blood and Tissue kit (Qiagen, Valencia, CA, USA) as per the manufacturer’s instructions.

## Results

We could identify a likely causative variant for the symptoms of the deceased children for 5 of 13 couples (38%, Table 1) and potentially causative variants in a novel disease candidate gene in 2 of 13 cases (15%, Table 1). Out of these 7 families we could confirm the variant in the homozygous or compound heterozygous state in the affected children of 4 families in which DNA sample of the deceased child/fetus was available (for genes *ACADVL, CTSD, PALLD, APAF1,* Table 1)*.*

**Fig. 1:**
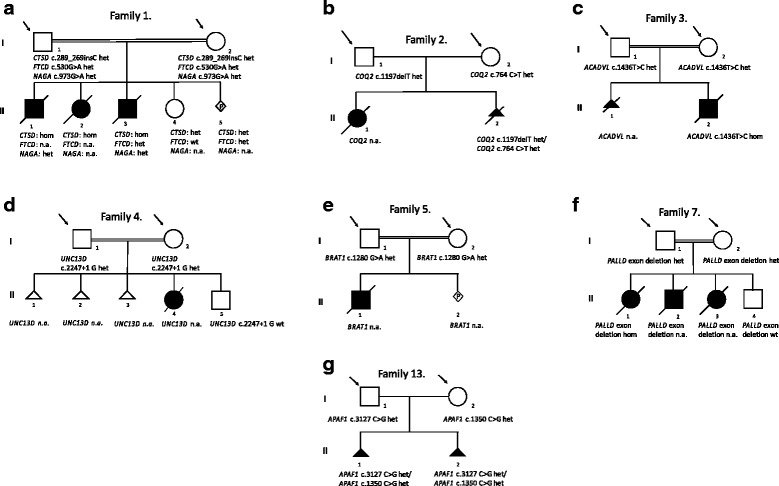
Pedigrees of the investigated couples with likely or possibly causative variants. **a**-**g** Pedigrees showing the sequence variants found in this study: arrow: index patients (unaffected parents) analyzed, full symbol: affected child/fetuses, n.a.: DNA was not available for investigation or prenatal diagnostics was declined

In **Family 1** the parents are first cousins (Fig. 1) and had lost three children (one girl and two boys) due to severe, refractory myoclonic epilepsy and respiratory insufficiency immediately after birth. Additional findings included ventriculomegaly, corpus callosum agenesis, severe microcephaly with marked cerebellar atrophy, massive reduction of brain weight and pachygyria. All three children died within weeks after birth [9]. The causative molecular defect, a homozygous deleterious variant in the *CTSD* gene (NM_001909: c.268_269insC, p.(Gln90Profs*50)) leading to infantile neuronal ceroid lipofuscinosis (CLN 10, OMIM #610127, [10]) had already been identified previously before coming to our clinic through targeted NGS analysis of their first two children [9]. Biochemical studies (absent cathepsin-D activity in fibroblasts of the first two children) performed by Steinfeld and coworkers underlined the pathogenicity of the *CTSD* variant [9]. We could confirm the same homozygous variant in DNA of the third deceased child. Because of the consanguinity we performed WES of the couple for additional potentially shared heterozygous variants before deciding on further reproductive options. Besides the known frameshift variant in the *CTSD* gene we could identify shared heterozygous variants in two more genes: *FTCD* and *NAGA* **(**Fig. 1 and Table 1**)**. Homozygous presence of the NM_001320412: c.530G > A, p.(Gly177Glu) variant in the *FTCD* gene leads to autosomal recessive glutamate formiminotransferase deficiency (OMIM: #229100) characterized by growth retardation, severe developmental delay and megaloblastic anemia [11]. The deceased children did not show any possible signs of this inborn error of metabolism. For the *FTCD* variant only the third child was investigated and was found to be heterozygous. The third shared heterozygous variant of the parents NM_000262: c.973G > A, p.(Glu325Lys) in the *NAGA* gene is described to lead to Schindler disease Type 1 when found homozygous in children [12]. However, a broad phenotypic spectrum has been reported in children homozygous for variants predicted to be pathogenic in the *NAGA* gene (and for the Glu325Lys variant as well), even including cases that do not show any neurological abnormality [13, 14]. The first and third affected child was shown to carry the heterozygous variant in the *NAGA* gene. In a fourth and fifth natural pregnancy of the couple prenatal diagnostics were performed. Out of the 3 heterozygous variants for potential autosomal recessive diseases in the offspring we found the *CTSD* and *FTCD* variants eligible for prenatal testing. We did not find the *NAGA* c.973G > A, p.(Glu325Lys) variant eligible for use in prenatal testing. In the fourth and fifth pregnancies of the couple the developing fetuses were tested to be heterozygous carriers of the *CTSD* variant. The fourth child was wildtype for the *FTCD* gene while the fifth fetus was heterozygous. No intrauterine seizures were observed in either pregnancy. The fourth child had an uneventful neonatal period, while the fifth pregnancy is still ongoing.

**Family 2 (**Fig. 1) is a non-consanguineous German couple who had lost their first child at the age of 3 days due to severe congenital lactic acidosis. Targeted NGS analysis of the couple revealed rare heterozygous potentially deleterious variants (NM_015697: c.1197delT, p.(Asn401Ilefs*15) in one partner and NM_015697: c.764C > T, p.(Pro255Leu) in the other partner) in the *COQ2* gene **(**Table 1**)**. Sanger sequencing confirmed heterozygosity of the parents. Unfortunately, no material was available from the deceased daughter for confirmation of compound heterozygosity. However, the child’s phenotype resembled that associated with loss of function of the *COQ2* gene (primary coenzyme Q10 deficiency, OMIM #607426, [15]). Although compound heterozygosity was not confirmed in the affected daughter we found the Class 4 and Class 5 variants of the parents eligible for prenatal diagnostics in the second pregnancy. Unfortunately, the fetus was shown to be compound heterozygous for the two *COQ2* variants and the pregnancy was terminated. The couple opted for PGD in the future.

The parents in the third family (**Family 3,** Fig. 1) were first cousins from Saudi-Arabia who had had a pregnancy termination due to ultrasound abnormalities in their first pregnancy and had lost their second child at the age of 5 months due to severe hypertrophic cardiomyopathy and sudden infant death syndrome. We identified the heterozygous variant NM_001033859: c.1436 T > C, p.(Pro479Leu) in the *ACADVL* gene in both parents associated with very long-chain acyl-CoA dehydrogenase deficiency (OMIM #201475). Sanger sequencing confirmed heterozygosity of both parents and homozygosity in the paraffin embedded sample of their deceased son **(**Table 1**)**. This variant has not been reported in the literature or listed in available databases. Homozygous or compound heterozygous mutations in the *ACADVL* gene, however, have been described to cause deficiency of the very long-chain acyl-CoA dehydrogenase (VLCAD-deficiency, OMIM# 201475) leading to, among other, hypertrophic cardiomyopathy, sudden cardiac death, hepatomegaly, hypotonia and lethargy [16]. A retrospective assessment of the dried blood spot carnitine ester profile of the deceased child was consistent with the biochemical diagnosis of VLCAD-deficiency. We found the Class 4 variant in the *ACADVL* gene eligible for prenatal diagnostics and reproductive options were offered to the couple.

A consanguineous couple from Egypt (first cousins, **Family 4,** Fig. 1**)** who had lost a child with clinically diagnosed severe lymphohistiocytosis after three miscarriages in early pregnancies displayed a rare heterozygous splice site variant in the *UNC13D* gene (NM_199242:c.2447 + 1G > T, p.?), associated with familial hemophagocytic lymphohistiocytosis-3 (Table 1). The rare splice site variant has not been reported in the literature or in available databases. The prediction tool Human Splicing Finder v.3 predicts the total loss of the 5‘-Splice-Donor-site, which in turn may lead to exon skipping of exon 25 of the *UNC13D* gene. Sanger sequencing confirmed heterozygosity in the parents. The affected girl had shown intrauterine growth deficiency and at the age of 3 weeks had developed a hyperinflammatory syndrome with hepatosplenomegaly, peritonitis, fever and systemic inflammation. She had died at one month of age. No material from the deceased child was available. Homozygous or compound heterozygous mutations in the *UNC13D* gene have been described to cause familial hemophagocytic lymphohistiocytosis type 3 (FHL3; OMIM: #608898). The disease is characterized by hyperinflammation with excessive activation of T lymphocytes and macrophages leading to hepatomegaly, liver function abnormalities, pancytopenia and coagulation defects [17], exactly the symptoms observed in the couple’s deceased daughter. The couple had a healthy son born one year after the affected daughter who was wildtype for the parental variant. We found the Class 4 variant in the *UNC13D* gene eligible for prenatal diagnostics in future pregnancies.

In a multiple consanguineous couple (first cousins and parents of the couple are also first cousins) from Saudi-Arabia (**Family 5,** Fig. 1) who had lost one child at 11 months of age due to an undiagnosed neurodegenerative disease resembling a mitochondriopathy with muscular hypotonia, later spasticity, severe intractable epilepsy, epileptic encephalopathy with respiratory insufficiency and degeneration of the basal ganglia our targeted NGS panel revealed a rare heterozygous missense variant in the *BRAT1* gene (NM_152743: c.1280G > A, p.(Arg427Gln)) in both parents (Table 1). Sanger sequencing confirmed the heterozygosity in the parents, but unfortunately, no DNA was available from the deceased child. This variant has not been reported in the literature or in available databases, however, it was predicted to be pathogenic applying various prediction tools. Homozygous or compound heterozygous mutations in the *BRAT1* gene have been described to cause lethal neonatal rigidity and multifocal seizures syndrome (RMFSL, OMIM #614498) leading to, among other, progressive postnatal microcephaly, muscular hypotonia, developmental delay, spasticity and rigidity, delayed myelination, degeneration of the basal ganglia, therapy-refractory epilepsy and in the end stage to a severe epileptic encephalopathy [18]. The reported symptoms overlapped with the available phenotypic data of the RMFSL syndrome. In addition, a recent publication by Horn et al. [19] showed that *BRAT1* mutations are associated with mitochondrial dysfunction and thus the described phenotype of the deceased child could well have resembled a mitochondriopathy. Thus we found the Class 4 variant eligible for prenatal diagnostics. In a following spontaneous pregnancy the couple was offered prenatal testing but did not wish to undergo invasive prenatal diagnostics.

In the other families (8/13) no shared causative variant in a known pathogenic gene or no variants in the same known gene were identified by targeted next generation sequencing **(**Tables 1**)**. In two out of those 8 families (Families 7 and 13) further analysis (microarray- and WES analysis) was completed, while in two further families (Families 10, 12) WES is still pending. The other families (Families 6, 8, 9, 11) did not consent to further studies. In families 7 and 13, in which no causative variants in established disease genes (OMIM) were found and further analysis is completed, we identified biallelic sequence or copy number variants in presumably novel candidate disease genes **(**Table 1, Additional file 2: Table S2**)**.

In one consanguineous family (first cousins) of Turkish origin (**Family 7,** Fig. 1) who had lost three children with a severe lissencephaly type 2 malformation in the neonatal period genome-wide microarray-analysis revealed a heterozygous exon 1 deletion of the *PALLD* gene (NM_001166108, OMIM *608092) in both parents. A quantitative real-time PCR assay confirmed heterozygosity in the parents and in the available DNA from the deceased first child homozygosity for the deletion. The only unaffected child of the family did not carry the *PALLD* deletion. Germline heterozygous variants in the *PALLD* gene have only been described in a few families with susceptibility to pancreatic cancer (OMIM #606856, [20]). However, functional studies show a significant role of the palladin gene in neuronal migration [21], thus, a potential causative role of a homozygous loss-of-function of this gene in the development of lissencephaly can be assumed. Analysis of the two other deceased children in the family is still pending due to the difficulty of available DNA. Unfortunately, at this point, no prenatal diagnostics can be offered to the family.

A second non-consanguineous German couple (**Family 13,** Fig. 1) was investigated because of two miscarriages in the 11th week of gestation due to anencephaly in both fetuses. After our targeted panel diagnostics (430 genes, for the list of genes see Additional file 1: Table S1.) did not reveal any causative alteration we performed whole exome sequencing in the couple and identified rare heterozygous potentially deleterious variants (NM_181861: c.1350C > G, p.(Cys450Trp) in one partner and c.3127C > G, p.(His1043Asp) in the other partner) in the *APAF1* gene (OMIM *602233). Sanger sequencing confirmed heterozygosity in both parents and compound heterozygosity in the available DNA of the affected fetuses from both pregnancies with anencephaly (Table 1). The *APAF1* gene encodes for the apoptotic protease inhibitor factor 1 that has been shown to play an essential role in mitochondrial pathways of apoptosis and brain development assembling into an oligomeric apoptosome, which is responsible for activation of procaspase-9 and maintenance of the enzymatic activity of processed caspase-9 [22, 23]. Although no human disease has been associated so far with this gene, mouse knock-out studies revealed an essential function of the gene in neurogenesis with full knock-outs showing anencephaly [24]. Therefore, a potential causative role of a biallelic mutation of this gene in neural tube development cannot be excluded. At this point, however, the variants can only be classified as Class 3 and thus are not eligible for PGD.

Applying WES in consanguineous couples for carrier screening we could detect 3–4 shared variants in the same gene on average. According to our guidelines we reported all variants to the couples that are classified as Class 3–5 variants [8]. However, we discussed with the couples that prenatal diagnosis or preimplantation genetic diagnosis can only be carried out for Class 4 and 5 variants.

## Discussion

We carried out carrier screening using a targeted next-generation sequencing approach and WES as further analysis in consanguineous and non-consanguineous couples at increased risk for autosomal recessive disorders. Increased risk was determined due to a positive family history, meaning at least one deceased child with a rare undiagnosed disease or because of multiple affected fetuses with congenital anomalies.

A likely causative variant for the symptoms of the deceased children was identified in 5 out of the 13 couples investigated (38% overall, 4/8 consanguineous and 1/5 non-consanguineous couples, Table 1) and potentially causative variants in novel candidate genes in 2 additional couples. In 4 out of these 7 families we had access to DNA material from a deceased child/fetus and could confirm the presence of the variant in the homozygous or compound heterozygous state (for genes *ACADVL, CTSD, PALLD, APAF1,* Table 1)*.*

Identifying disease causing heterozygous mutations with recessive transmission in carriers is far more difficult than identifying homozygous disease-causing mutations in affected patients, which is also reflected by the low yield of carrier identification compared to the diagnostic yield in affected patients using established techniques [6]. Therefore, as long as there are no affected homozygotes in the respective families documenting that the respective mutation is indeed disease-causing, caution has to be used in offering prenatal diagnostics to couples carrying the same mutation as heterozygotes, at least if the mutation has not been described as disease-causing before. In our analysis we could identify likely disease-causing or pathogenic sequence variants in 5 out of our initial cohort of 13 couples and potentially disease-causing variants in further 2 couples. Subsequent prenatal diagnosis or preimplantation genetic diagnosis has been performed or is in progress based on the results of four of the five couples (Families 1–4, Table 1) in which likely pathogenic or pathogenic sequence variants (Class 4 or 5) predicting high recurrence risks have been identified (for variants *CTSD, FTCD, COQ2, ACADVL, UNC13D*). In two of the couples with likely disease-causing variants (Class 4) the variants were confirmed to be homozygous in the available DNA sample of the deceased children, thus prenatal testing or preimplantation genetic diagnostics options were offered to the families (Families 1 and 3 for variants *CTSD, FTCD, ACADVL*). In three further couples, either established disease-causing mutations were found at least in one parent, while the other carried a yet unreported likely pathogenic variant, or both parents were found to be carriers of the same deleterious yet unreported variant. In these cases, there were strong similarities between the clinical diagnosis of the deceased children and the phenotypic spectrum associated with the identified genes. Therefore, we decided to offer prenatal diagnostics and/or preimplantation genetic diagnosis to those three couples (Families 2, 4, 5 for variants *COQ2, UNC13D, BRAT1*) based on the identified gene defects. One of those couples (Family 5) did not wish to undergo prenatal diagnosis in a following spontaneous pregnancy.

In 8 out of 13 couples no variants in known pathogenic genes were identified by targeted panel diagnostics. There are many possibilities that may have accounted for the cause of death in those families, including a gene defect not targeted by our panel, variants in regulatory regions uncovered by exon enrichment, but also non-genetic causes cannot be excluded even in consanguineous families. Although our panel of selected genes includes the majority of autosomal recessive genes with a known congenital malformation syndrome, metabolic defect or severe neurodevelopmental disorder, whole exome sequencing provides the ultimate test for the detection of carrier status in consanguineous couples [25]. Out of the 8 couples in which no causative diagnosis could be established 4 consented to undergo further microarray- and WES analysis. In one of the two couples (Family 13) in which whole exome sequencing was completed possibly causative heterozygous variants in a gene not yet associated with human disease were identified and confirmed to be compound heterozygous in the two affected fetuses. In two further families WES is still pending.

Furthermore, our diagnostic pipeline did not allow a sufficient detection of copy number variations in the investigated genes. Therefore, we additionally performed genome-wide microarray analysis in 4 non-consanguineous couples of which only one partner carried a potentially deleterious sequence variant compatible with the phenotype of the deceased child but no variant was found in the partner. Furthermore, we also performed microarray analysis in two consanguineous couples in which no variants were found in disease genes. In one of these consanguineous couples (Family 7) microarray analysis identified a presumably disease-causing heterozygous exon deletion in a candidate disease gene that was confirmed to be homozygous in the first affected child. Novel disease genes will be described in detail in separate.

Finally, several issues could lead to failure in detecting disease causing variants in parents using the methods we describe here, including technical issues such as an insufficient coverage of some genes, deep intronic or splice site variants not detected by the applied pipeline, trinucleotide expansion diseases or an incorrect hypothesis for the mode of inheritance.

Rare and undiagnosed autosomal recessive diseases frequently occur in the offspring of consanguineous couples. Current routine diagnostic procedures often fail to identify the underlying genetic defect. Many studies have shown that NGS panel diagnostics or diagnostic exome sequencing can be a powerful tool for the detection of carrier status in consanguineous or even non-consanguineous couples with positive family history suggestive of a recessive disorder [26]. The identification of a causative variant(s) can be used for the estimation of the risk for affected offspring, for family planning and enables informed reproductive decision-making for the affected families [3, 25, 27]. A recent study has shown, however, that practical challenges in genetic counseling should not be underestimated and should be addressed carefully in couples before implementing expanded carrier screening in the clinical setting [28]. Consanguineous marriages occur in significant numbers around the world, accounting for 20%–50% in several regions of the Middle East and the Mediterranean basin but also increasingly affecting populations in Western European countries [29, 30]. Children born to consanguineous couples are at increased risk of presenting with congenital anomalies [26, 31]. Even for consanguineous couples with a negative family history, prospective carrier screening may be useful to minimize the increased basal risk of 6–10% for giving birth to a child with a congenital malformation or condition.

Identified variants in our cohort enabled informed reproductive decision-making in five affected families from our study in which a causative diagnosis was established and are now used for prenatal diagnostics and preimplantation genetic diagnosis in four out of those five families. In the affected offsprings of Families 7 and 13 homozygous and compound heterozygous variants in novel candidate genes were identified. Since to our current knowledge these variants cannot be classified higher than Class 3 variants [8] to this point no PGD or prenatal diagnostics can be offered to those families in future pregnancies. Further publications of the association of these candidate genes and a similar clinical phenotype in patients or further functional studies need to be done before the variant can be classified to a higher pathogenicity level eligible for prenatal diagnostics or PGD.

## Conclusion

Our data show that NGS based gene panel sequencing of selected genes involved in lethal autosomal recessive disorders is an effective tool for carrier screening in parents and for the identification of recessive gene defects in families that have experienced early child death and/or multiple abortions.

## Additional files

Additional file 1: Table S1. List of the 430 genes investigated in the couples. Additional file listing the 430 genes for severe AR and XL disorders investigated in the couples (XLS 142 kb)

Additional file 2: Table S2. Additional variants in the couples. Additional file describing additional possibly pathogenic variants in the couples and sequencing quality of the variants (DOCX 21 kb)

## Abbreviations

AR: autosomal recessive
C: consanguineous
EUCS: expanded universal carrier screening
NC: non-consanguineous
NGS: next generation sequencing
PGD: preimplantation genetic diagnosis
