# Supplementary material for: Targeted next-generation sequencing analysis in couples at increased risk for autosomal recessive disorders
Source: Orphanet J Rare Dis. 2018 Jan 26;13:23. doi: 10.1186/s13023-018-0763-0 (PMC5787287; doi:10.1186/s13023-018-0763-0)
Supplement: Supplementary file 2 — Additional variants in the couples. Additional file describing additional possibly pathogenic variants in the couples and sequencing quality of the variants (DOCX 21 kb) [file 13023_2018_763_MOESM2_ESM.docx]

**Table S2: Identified variants in the couples and sequencing quality of the variants**

| **Family ID** | **Con-**  **sang-uinity** | **Shared high-quality heterozygous variants in the couple/variants in the same gene** | **CADD score** | **Disease (OMIM)**  **Increased risk for autosomal recessive disorder** | **Additional high-quality variants identified in one parent** | **CADD score/**  **read depth*** | **Disease (OMIM)**  **Increased risk for autosomal recessive disorder** | **Coverage of the gene in the partner**  **Cov. >= 10** |
| --- | --- | --- | --- | --- | --- | --- | --- | --- |
| 1 | yes | *CTSD* (*116840)  NM_001909: c.268_269insC p.(Gln90Profs*50)  *FTCD* (*606806)  NM_001320412: c.530G>A p.(Gly177Glu)  *NAGA* (*104170)  NM_000262: c.973G>A, p.(Glu325Lys) | n.a.  25.8  32.0 | Ceroid lipofuscinosis,  type 10 (#610127)  Glutamate formiminotrans-ferase deficiency (#229100)  Schindler disease (#609241) | *KCNMA1* (*600150) NM_001161352:  c.2261G>A, p.(Arg754His)  *TECTA* (*6025749)  NM_005422: c.5836T>C, p.(Tyr1946His)  *CFTR* (*602421)  NM_000492: c.202A>G, p.(Lys68Glu) | 13.3/  39,38  29.2/  103,94  23.8/  41,52 | ?Cerebellar atrophy, developmental delay, and seizure (#617643)  Deafness, autosomal recessive 21(#603629)  Cystic fibrosis (#219700) | 99%  93%  99% |
| 2 | no | *COQ2* (*609825)  mother: NM_015697: c.1197delT p.(Asn401Ilefs*15)  father: c.764C>T p.(P255L) | 23.7  29.0 | Coenzyme Q10 deficiency, primary, type 1 (#607426) | *COL6A3*(*120250)  NM_004369: c.5554T>A, p.(Phe1852Ile) | 23.1/  31,17 | Ullrich congenital muscular dystrophy 1 (#254090) | 83% |
| 3 | yes | *ACADVL* (*609575) NM_001270447: c.1274T>C, p.(L425P) | 27.4 | VLCAD deficiency, (#201475) | *DSP* (*125647)  NM_004415: c.3551G>A, p.(Arg1184Gln) | 24.9/  147,  104 | Cardiomyopathy, dilated, with woolly hair and keratoderma (#605676) | 95% |
| 4 | yes | *UNC13D* (*608897)  NM_199242: c.2447+1G, p.? | 27.4 | Familial hemophagocytic lymphohistiocyto-sis type 3 (#608898) | *SPTBN2* (*604985)  NM_006946: c.1456G>A, p.(Ala486Thr)  *AARS* (*601065)  NM_001605: c.1172G>A, p.(Arg391His) | 24.0/  40,54  34.0/  67,68 | Spinocerebellar ataxia, autosomal recessive 14 (#615386)  Epileptic encephalopathy, early infantile, 29 (#616339) | 94%  99% |
| 5 | yes | *BRAT1* (*614506)  NM_152743: c. 1280G>A, p.(Arg427Gln)  *FLNB* (*603381)  NM_001164317: c.2789 C>T, p.Pro930Leu | 32.0  34.0 | Rigidity and multifocal seizure syndrome (#614498)  Spondylocarpo-tarsal synostosis syndrome (#272460) | *CACNA1D* (*)  NM_000720: c.2527G>A, p.(Val843Met) | 23.2/  35,24 | Sinoatrial node dysfunction and deafness (#614896) | 99% |
| 6 | no | none |  |  | none for AR disease |  |  |  |
| 7 | yes | *PALLD* (*608092) NM_001166108: Exon 1 deletion | n.a. | No OMIM disease entry  mouse: migration defect [28] | *NDUFS1* (*157655)  NM_001199984: c.1712G>A, p.(Arg571Gln)  *CLCN1* (*118425)  NM_000083: c.2864A>T,  p.(Glu955Val) | 24.5/  63,63  28.5/  115,  137 | Mitochondrial complex I deficiency (#252010)  Myotonia congenita, recessive (#255700) | 99%  97% |
| 8 | yes | none |  |  | none for AR disease |  |  |  |
| 9 | no | none |  |  | none for AR disease |  |  |  |
| 10 | yes | none |  |  | none for AR disease |  |  |  |
| 11 | no | none |  |  | *SYNE1* (*608441) NM_182961: c.25091C>T, p.(Pro8364Leu) | 34.0/  24,32 | Spinocerebellar ataxia, autosomal recessive 8 (#610743) | 93% |
| 12 | yes | none |  |  | *CLCN2* (*600570)  NM_004366:  c.1930C>T, p.(Arg644Cys)  *PLEC* (*601282) NM_201380:  c.6785C>T, p.(Ala2262Val) | 35.0/  27,25  18.87/  39,21 | Leukoencephalo-pathy with ataxia (#615651)  Muscular dystrophy, limb-girdle, type 2Q (#613723) | 98%  99% |
| 13 | no | *APAF1* (*602233)  mother: NM_181861: c.1350C>G p.(Cys450Trp)  father: c.3127C>G p.(His1043Asp) | 23.1  25.7 | No OMIM disease entry  mouse model: anencephaly, neurogenesis defect [31] | *ABCC6* (*603234)  NM_001171: c.3787G>A, p.(Gly1263Arg)  *CHRND* (*100720)  NM_000751: c.380A>G, p.(Tyr127Cys) | 32.0/  44,39  24.0/  9,15 | Arterial calcification, generalized, of infancy, 2 (#614473)  Multiple pterygium syndrome, lethal type (#253290) | 89%  100% |

**Abbreviations**: n.a.: not applicable, CADD: Combined Annotation Dependent Depletion score, *: read depth is given for the wild-type allele

first and then for the mutated allele
